# Quality of life and cost consequence of delays in endovascular treatment for acute ischemic stroke in China

**DOI:** 10.1186/s13561-021-00352-w

**Published:** 2022-01-06

**Authors:** Weiyi Ni, Wolfgang G. Kunz, Mayank Goyal, Lijin Chen, Yawen Jiang

**Affiliations:** 1 Department of Pharmaceutical and Health Economics, University of Southern California, Los Angeles, California USA; 2 Department of Radiology, University Hospital, LMU Munich, Munich, Germany; 3 Departments of Radiology and Clinical Neurosciences, University of Calgary, Calgary, Alberta Canada; 4 Chronic Disease Research Institute, School of Public Health, Zhejiang University School of Medicine, Hangzhou, Zhejiang China; 5 School of Public Health (Shenzhen), Sun Yat-sen University, Room 215, Mingde Garden #6, Sun Yat-sen University, 132 East Outer Ring Road, Panyu District, Guangzhou, Guangdong China

**Keywords:** Cost-effectiveness, Acute ischemic stroke, Endovascular treatment

## Abstract

**Background:**

Although endovascular therapy (EVT) improves clinical outcomes in patients with acute ischemic stroke, the time of EVT initiation significantly influences clinical outcomes and healthcare costs. This study evaluated the impact of EVT treatment delay on cost-effectiveness in China.

**Methods:**

A model combining a short-term decision tree and long-term Markov health state transition matrix was constructed. For each time window of symptom onset to EVT, the probability of receiving EVT or non-EVT treatment was varied, thereby varying clinical outcomes and healthcare costs. Clinical outcomes and cost data were derived from clinical trials and literature. Incremental cost-effectiveness ratio and incremental net monetary benefits were simulated. Deterministic and probabilistic sensitivity analyses were performed to assess the robustness of the model. The willingness-to-pay threshold per quality-adjusted life-year (QALY) was set to ¥71,000 ($10,281).

**Results:**

EVT performed between 61 and 120 min after the stroke onset was most cost-effective comparing to other time windows to perform EVT among AIS patients in China, with an ICER of ¥16,409/QALY ($2376) for performing EVT at 61–120 min versus the time window of 301–360 min. Each hour delay in EVT resulted in an average loss of 0.45 QALYs and 165.02 healthy days, with an average net monetary loss of ¥15,105 ($2187).

**Conclusions:**

Earlier treatment of acute ischemic stroke patients with EVT in China increases lifetime QALYs and the economic value of care without any net increase in lifetime costs. Thus, healthcare policies should aim to improve efficiency of pre-hospital and in-hospital workflow processes to reduce the onset-to-puncture duration in China.

## Background

The healthcare system in China faces a hefty burden of stroke. The age-standardized prevalence of stroke in China were 1114.8 per 100, 000 individuals. Stroke is also one of the leading causes of death in China, accounting for 1.57 million deaths in 2018. Among all type of strokes including acute ischemic stroke (AIS), intracerebral hemorrhage and subarachnoid hemorrhage, AIS took almost 82% [1].

Clinical efficacy of EVT in the treatment of AIS has been demonstrated compared to intravenous thrombolysis (IVT) in improving mortality rates and functional outcomes among patients with AIS, and further influences post-stroke care in the long-term [2–7]. According to previous studies the outcomes of EVT are time-dependent and decline with increasing delay between stroke onset and initiation of EVT [8]. As such, the treatment guidelines in China recommend EVT in AIS patients within 6 h of symptom onset [1].

However, significant delays of treatment to AIS patients are present in most of healthcare systems and has a fundamental negative impact on outcomes from both clinical and economic perspective. Recently, two studies investigated the health-related quality of life and cost consequences of delays for stroke patients in the US and Singapore, respectively. The US-based study demonstrated that every hour of treatment delay in EVT reduced a patient’s quality-adjusted life years (QALY) by 0.77 [9] and the Singapore study showed AIS patients treated with EVT at early time window had higher quality-adjusted life year (QALY) and less long-term healthcare costs [10]. Similar study had also been conducted in Italy, which documented the cost-effectiveness of EVT for the treatment of AIS patients [11]. However, such evidence is still lacking among Chinese AIS patients. Since cost-effectiveness profiles not only depend on treatment efficacy but also vary across institutional contexts such as local costs of procedures and other treatments, the conclusions from studies conducted in other countries and regions are not directly applicable to the Chinese setting. Moreover, as the Hospital Quality Monitoring System data showed, the rate of AIS patients treated with EVT in 2018 was only 2.81%, which is mainly because of the higher cost of EVT comparing to the alternative treatments. Hence, it is meaningful to conduct a cost-effectiveness analysis of delay of EVT in China so that the decision makers can have a better insight on the consequence of EVT. The purpose of the present study was to analyze the impact of delay in EVT on healthcare costs and QALYs on the population in Chinese and determine its cost-effectiveness within different time windows of symptom onset.

## Methods

### Model overview

A Markov health state transition model was constructed using (TreeAge Pro 2018, TreeAge, Willliamstown, MA) to compare six treatment time windows among a base-case cohort of patients with AIS aged 66 [1]. Outcomes within treatment initiation time windows of 61–120 min, 121–180 min, 181–240 min, 241–300 min, 301–360 min, and 361–420 min from onset were simulated over a lifetime horizon. Incremental cost-effectiveness ratios (ICER) defined as incremental costs/QALY and net monetary benefit (NMB) were calculated to evaluate cost-effectiveness. We used a willingness-to-pay (WTP) threshold of ¥71,000 per QALY (US$10,280/ QALY), which is the 2019 gross domestic product (GDP) per capita in China [12].

### Model structure and inputs

**Fig. 1:**
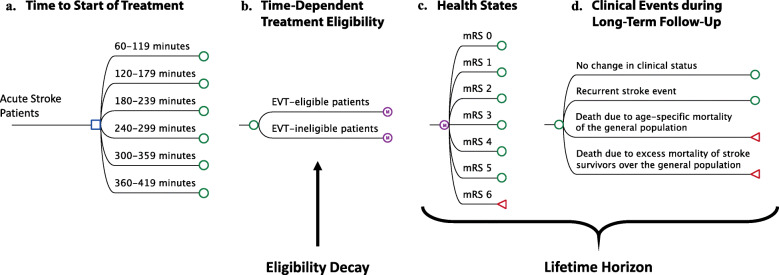
Model Structure Acute ischemic stroke patients in China entered the model-based analysis (**A**), received either EVT or non-EVT treatment based on the eligibility rate at different treatment initiation time windows (**B**), and entered a health state based on the modified Rankin Scale (mRS) score at 90 days (**C**). During each one-year cycle of Markov model, patients remained in the same health state, experienced a recurrent stroke, or died from either age-specific mortality or excess mortality due to stroke (**D**)

A short-term decision tree model was created to analyze acute healthcare costs during index AIS stroke hospitalization. Figures 1A-D detail the structure of the model chronologically. We assigned patients to receive either EVT or non-EVT treatment based on the probability of eligibility for EVT at different treatment initiation time windows. Treatment eligibility probabilities for the overall study population and patient subgroups were extracted from the HERMES collaboration’s meta-analysis of patient-level data from the five major randomized controlled trials (RCTs) (MR CLEAN, ESCAPE, REVASCAT, SWIFT PRIME, and EXTEND IA) [2]. EVT eligibility was assumed as 100% for patients presenting within 2 h of symptom onset, and then decreased by 3% every 30-min delay; this was a conservative assumption based on expert consensus review of the existing literature (Table 1) [2]. To account for patients who received IVT, the acute treatment costs implied in both EVT and non-EVT strategies were adjusted by the percentage of patients receiving IVT from clinical trials [8]. After treatment assignment, patients entered 1 of the 7 possible health states according to the degree of disability as assessed by the modified Rankin Scale (mRS) score of 0 to 6. The mRS score was further used to calculate healthcare costs.

**Table 1:** Clinical Input Parameters

Model Input	Base-Case Value	Range for Sensitivity Analysis	Reference
**Initial Probabilities**			
For each health state mRS 0–6 among EVT-treated patients	90-day mRS distribution for different times to EVT	Adjusted by distribution according to sample size	HERMES Data [2, 8]
For each health state mRS 0–6 among EVT-ineligible patients	90-day mRS distribution of ASPECTS 0–5 control arm	Adjusted by distribution according to sample size	HERMES Data [2, 8]
**EVT Eligibility by Time**			
61–120 min	1	0.90–1.00	Boulouis et al. [13] and expert consensus
121–180 min	0.94	0.84–1.00	
181–240 min	0.88	0.78–0.98	
241–300 min	0.82	0.72–0.92	
301–360 min	0.76	0.66–0.86	
**IVT Eligibility**			
EVT Patients	0.83	0.82–084	HERMES Data [2]
Non-EVT Patients	0.88	0.87–0.89	
**Transition Probabilities**			
Recurrent stroke rate	Time-dependent values	0.044–0.082	Pennlert et al. [14]
Annual death rate of population	Age-dependent values	N/A	China Life Table [15]
**Death hazard ratio by mRS, relative to general age-matched population**			
mRS 0	1.54	1.21–1.84	Hong et al. [16]
mRS 1	1.54	1.21–1.84	
mRS 2	2.18	1.58–1.69	
mRS 3	3.18	1.58–1.69	
mRS 4	4.56	2.37–3.03	
mRS 5	6.56	3.83–6.44	
**mRS Distribution**			
mRS after recurrent stroke	90-day mRS distribution of HERMES control arm	Adjusted by distribution according to sample size	HERMES Data [2, 8]

*EVT* endovascular therapy, *mRS* modified Rankin Scale, *ASPECTS* Alberta Stroke Program Early CT Score, *HERMES* Highly Effective Reperfusion evaluated in Multiple Endovascular Stroke Trials

Based on simulated 90-day mRS in the short-term model, patients entered the long-term Markov model to simulate outcomes over a lifetime horizon, using a 1-year cycle-length. The combination of a short-term model with a long-term model combined the data from the short-term outcomes derived from recent RCTs with additional data from long-term observational studies.

During each cycle of the Markov model, patients could remain in the same health state, experience a recurrent stroke, or die from either age-specific mortality or excess mortality due to history of stroke. Given that the rate of recurrent stroke rate is age-dependent, we implemented yearly recurrent stroke rates following the index stroke based on a stroke registry [14]. The total healthcare costs for each patient were the sum of the short-term healthcare costs after index AIS and lifetime healthcare costs. Recurrent stroke rates with corresponding mRS scores were obtained from the study by Pennlert et al. [14]. The age-specific death rate was drawn from the China Life Table [15]. Excess mortality risk due to stroke was incorporated in the model as the hazard rate ratio for each mRS health state obtained from a global clinical study [16], relative to age-matched controls without AIS in the general population (Table 1).

### Costs

All costs are reported in 2019 Chinese Yuan (¥). Both the short-term and long-term healthcare costs by mRS score were from China National Stroke Registry (CNSR) [17]. The costs of EVT and IVT were based on Endovascular therapy for Acute Ischemic Stroke Trial (EAST) and Thrombolysis Implementation and Monitor of Acute Ischemic Stroke in China (TIMS-CHINA) [17]. All costs were discounted by 3% each year (Table 2) [19].

**Table 2:** Healthcare Costs and Utilities

	Costs/Utility	Lower Boundary	Upper Boundary	Reference
**Acute 90-day Healthcare Costs; by 90-day mRS**				
mRS 0–2	¥11,314	¥11,147	¥11,483	CNSR [17]
mRS 3–5	¥15,448	¥15,109	¥15,792	
mRS 6	¥12,513	¥11,499	¥13,594	
**Long-Term Annual Healthcare Costs; by 90-day mRS**				
mRS 0–2	¥8310	¥8052	¥8573	CNSR
mRS 3–5	¥12,771	¥11,499	¥13,594	
**Additional Cost of IVT**	¥68,436	¥58,864	¥79,115	EAST [17]
**Additional Cost of EVT**	¥12,579	¥11,877	¥13,310	CNSR, TIMS-CHINA [17]
**Cost of Recurrent Stroke**	¥15,448	¥15,109	¥15,792	EAST [17]
**Utilities; by 90-day mRS**				
mRS 0	0.92	0.88	0.96	Ali et al. [18]
mRS 1	0.84	0.81	0.87	
mRS 2	0.74	0.70	0.78	
mRS 3	0.58	0.53	0.63	
mRS 4	0.37	0.32	0.42	
mRS 5	0.15	0.11	0.19	

All costs are in Chinese Yuan. *EVT* endovascular treatment, *IVT* intravenous thrombolysis, *mRS* modified Rankin Scale

### Utilities

Cumulative outcomes of alternative therapeutic courses were measured by quality-adjusted life years (QALYs). Utility weights were derived from a study by Ali et al. including stroke patients from Asian countries [18]. Utility values ranged from 0.15 for patients with an mRS of 5 to 0.92 for those with an mRS of 0 (Table 2). All QALYs were discounted at an annual rate of 3% [19].

### Cost-effectiveness analysis

Cost-effectiveness was compared in terms of ICERs and INMB. The ICER was calculated as incremental costs divided by incremental QALYs. INMB rearranges the ICER and incorporates a WTP per QALY in China, which was set to S$71,000 per QALY. Generally, a positive INMB value suggests that the intervention should be adopted per the health system’s WTP threshold.

### Sensitivity analysis

We used deterministic sensitivity analyses to test the robustness of the model results. Deterministic one-way sensitivity analysis was performed to identify variables that significantly influence the modeled outcomes. Input ranges for deterministic sensitivity analysis were determined by the 95% confidence interval of the initial probabilities, utilities, and costs (Table 1 and Table 2**)**. A probabilistic sensitivity analysis was also undertaken to evaluate the robustness of base-case results in the presence of simultaneous variability of the input variables. We assumed that the costs followed a gamma distribution, death hazard ratio followed a log-normal distribution, and probabilities and eligibility rates followed a beta distribution. The simulation was run 10,000 times.

As a modeling exercise based on simulation, this study did not require Institutional Review Board approval as the input parameters for this modeling study were obtained from published literature and expert opinion, in which patient-identifiable information was not available. No primary clinical data were collected for this study. The study was reported according to the Consolidated Health Economic Evaluation Reporting Standards (CHEERS) statement [20].

## Results

### Base case analysis

Based on simulated outcomes from the six time windows in our model, EVT performed in the earlier time window was associated with more life-time QALYs and higher total healthcare costs. Based on the increasing order of simulated costs, EVT initiated within 301–360 min was associated an ICER of ¥15,712 in relation to the time window of 361–420 min. The time windows of 241–300 min, 181–240 min and 121–180 min were extendedly dominated by the time window of 61–120 min because the ICERs of the three time windows were larger than the ICER of the latter when comparing with the time window of 301–360 min. Moreover, the ICER of time window of 61–120 vs. 301–360 was ¥16,409. Hence, initiation of EVT between 61 and 120 min was most cost-effective among all the time windows when using once the GDP per capita as the threshold of WTP per QALY (Table 3). Each hour delay in initiating EVT resulted in an average loss of 0.45 QALYs and 165.02 healthy days (Table 4). Consequently, the average net monetary loss per hour due to delay in EVT treatment was estimated at ¥15,105 (Table 4).

**Table 3:** Results of Cost-Effectiveness Analyses of Time Delays in EVT Treatment

Time Window of EVT Initiation	Cost	Incremental Cost	QALY	Incremental QALY	ICER	INMB
361–420 min	¥120,285		2.45			
301–360 min	¥128,225	¥7940	2.95	0.51	¥15,712	¥27,938
241–300 min	¥137,671	¥9447	3.42	0.46	Extended domination	–
181–240 min	¥140,133	¥2462	3.57	0.15	Extended domination	–
121–180 min	¥150,357	¥10,224	4.19	0.62	Extended domination	–
61–120 min	¥158,616	¥8259	4.81	0.61	¥16,409	¥101,106

*EVT* endovascular therapy, *QALY* quality-adjusted life-years, *ICER* incremental cost-effectiveness ratio, *INMB* incremental net monetary benefit

**Table 4:** QALY, Healthy Life Days, and NMB Loss by Time

Items	Measurement
Average QALY per minute	0.0075
Average QALY per hour	0.45
Average Healthy Life days per minute (day)	2.75
Average Healthy Life days per hour (day)	165.02
Average NMB per minute (¥)	252
Average NMB per hour (¥)	15,105

### Sensitivity analysis

**Fig. 2:**
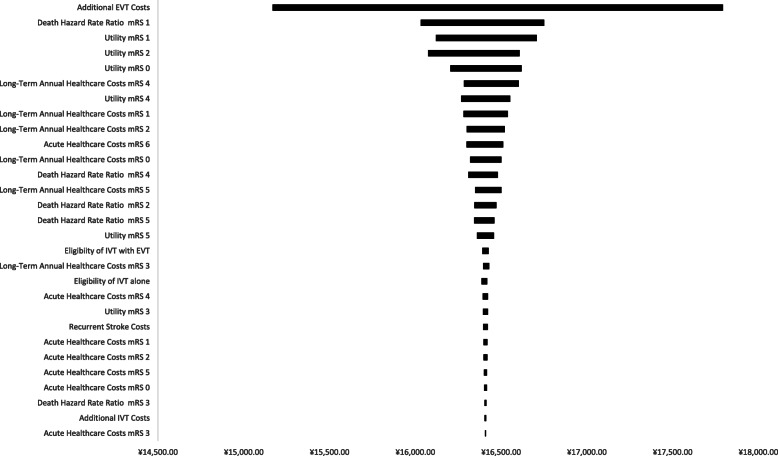
Deterministic One-Way Sensitivity Analysis: 61–120 min Subgroup vs. 301–360 min Subgroup The tornado graph indicates changes in the ICER as a result of deterministic one-way sensitivity analysis of the indicated model input parameters. EVT = endovascular therapy; mRS = modified Rankin Scale; IVT = intravenous thrombolysis. All costs are Chinese Yuan

The results of the deterministic one-way sensitivity analysis are presented in Fig. 2. The time windows of 61–120 min and 301–360 min were compared when the input parameters were varied within pre-specified ranges. The ICER comparison between the 61–120 min and 301–360 min time windows ranged from approximately ¥15,000 to ¥18,000 for all deterministic sensitivity results (horizontal bars in Fig. 2). Based on the once the GDP per capita WTP threshold, treatment initiated within 61–120 min of stroke onset was the cost-effective option in reference to the 301 to 360 min alternative. More, the outcomes demonstrated that ICER was most sensitive to the additional costs of EVT.

**Fig. 3:**
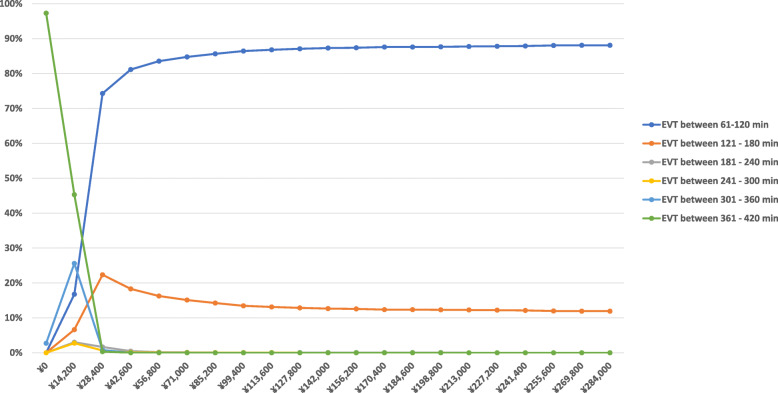
Cost-effectiveness acceptability curves The curves show the probabilities that each treatment time window is most cost-effective compared to the others over a range of willingness-to-pay thresholds

The probabilistic sensitivity analysis also illustrated that EVT treatment within 61–120 min after the stroke onset was the cost-effective strategy in 84.8% of simulations at the once the GDP per capita WTP threshold (Fig. 3). This increased to 87.8% at a WTP threshold of three times of GDP per capita at ¥213,000.

## Discussion

This study investigated the lifetime consequences of delayed initiation of EVT after stroke onset in terms of health-related quality of life and costs of EVT from the China healthcare perspective. Initiating EVT within 61–120 min after symptom onset was shown to be most cost-effective. We applied input parameters specific to the AIS patients in China, with both short- and long-term costs as well as utilities. The evidence of clinical benefit of EVT does not necessarily warrant its cost-effectiveness without considering its impact on healthcare costs, and it is essential for policymakers and clinicians to determine whether the benefits of EVT outweighs the higher cost of EVT compared to alternative treatments. The current study demonstrates that despite the higher short-term healthcare costs of EVT, EVT generated more QALYs, and is most cost-effective at earlier time windows after symptom onset.

Our findings are similar to those reported in cost-effectiveness studies of EVT in both the US and Singapore setting. In the US, each hour of delay in EVT resulted in an average loss of 0.77 QALYs and increased the healthcare cost by US$6173/QALY [9]. The Singapore study demonstrated earlier treatment with EVT was cost-effective, when the threshold of WTP per QALY was SG$36,500 [10]. Previous studies also showed with EVT and IVT was associated with lower disability at 90 days after stroke compared to IVT alone [8], with every of hour of delay in EVT reducing the absolute risk difference for good outcome by 6% [21]. However, those results could not be directly adopted to the China AIS patients, because the healthcare system and patient preferences to health states were different than either the US or Singapore. Hence, the current study is first to investigate the delay of EVT to AIS patient from China healthcare perspective to our knowledge.

The total healthcare costs associated with the 61–120 min subgroup were higher versus patients treated at later time points. This was driven by the greater proportion of patients eligible for EVT at the earlier time window and, therefore, a greater proportion of patients incurring costs of EVT relative to the less expensive alternatives. Patients with EVT have higher probability of improved functional outcome (lower mRS score), which is likely to reduce long-term costs associated with nursing home or home help relative to the alternative treatments, although the reduction in costs cannot fully offset the additional costs from EVT. Similar to our findings, Pan et al. reported EVT was associated with higher lifetime costs compared to IVT alone [17]. In spite of the greater initial treatment-related costs, EVT was found to be cost effective in previous cost-effectiveness studies performed in both western and eastern countries [17, 22–28].

Moreover, we simulated six different treatment windows in the current study to demonstrate the cost-effectiveness. In reality, clinicians may not be free to choose when to treat because the time from onset to puncture depends on many factors, such as patient transportation, accessibility of EVT at hospitals and affordability. Our findings that earlier treatment of EVT is more cost-effective may provide supportive evidence from the economic perspective to healthcare decision makers on improving the delay to perform EVT on AIS patients in China.

The current study has several limitations. First, annual healthcare costs for patient survived from AIS was applied for long-term costs. It is possible that the healthcare costs incurred in the first 2 years post-stroke was greater than those in subsequent years. However, this time-variant disease burden was not reflected in the current input parameters. To partially address this limitation, we performed a deterministic sensitivity analysis on annual healthcare costs. Second, the current model was constructed from the China healthcare perspective, which did not account for indirect costs related to potential productivity loss. However, given the majority of stroke population consisted of patients with advanced age, indirect costs would likely contribute far less than the direct healthcare costs in the present study. Moreover, similarly to other economic modeling studies, conclusions from the current study are region-specific and typically are not directionally applicable to other regions given specific medical costs, health utility preferences, and WTP thresholds vary. For example, the management and financing of healthcare facilities in European countries could be drastically different from those in China [29–33]. The direct costs of EVT spread over a wide range across countries as of $14,544 in the US [26], $3000 in Singapore [10] and $1980 in China. Future local adaptations to additional regions using the model presented herein would provide more specifics on local ICER values and a more precise local time window in which treatment with EVT is no longer considered cost-effective. Finally, preventive remedies to AIS may change the occurrence rate of AIS among Chinese population, which is not covered by current modeling. The cost-effectiveness of preventive treatments in China may worth an stand-alone study.

## Conclusions

This study indicates that performing EVT at earlier time windows is more cost-effective compared to initiating treatment at a later time after stroke onset from the China healthcare system perspective. Healthcare policies in China need to be implemented to improve efficiency of pre-hospital and in-hospital workflow processes to reduce the time to perform EVT on AIS patients.

## Data Availability

All model inputs related to this study can be found within the article and tables. The full TreeAge model has been provided for editorial and peer review and can be obtained from the corresponding author upon reasonable requests.

## Abbreviations

AIS: Acute ischemic stroke
CNSR: China National Stroke Registry
EVT: Endovascular therapy
GDP: Gross domestic product
ICER: Incremental cost-effectiveness ratio
INMB: Incremental net monetary benefit
IVT: Intravenous thrombolysis
mRS: modified Rankin Scale
QALY: Quality-adjusted life-year
RCT: Randomized controlled trial
TIMS-CHINA: Thrombolysis Implementation and Monitor of Acute Ischemic Stroke in ChinaWTP Willingness-to-pay
